# Enhanced Network Intrusion Detection System for Internet of Things Security Using Multimodal Big Data Representation with Transfer Learning and Game Theory

**DOI:** 10.3390/s24134152

**Published:** 2024-06-26

**Authors:** Farhan Ullah, Ali Turab, Shamsher Ullah, Diletta Cacciagrano, Yue Zhao

**Affiliations:** 1 School of Software, Northwestern Polytechnical University, Xian 710072, China; farhankhan.cs@yahoo.com (F.U.); aliturab@nwpu.edu.cn (A.T.); 2 Division of Computer Science, University of Camerino, 62032 Camerino, Italy; diletta.cacciagrano@unicam.it; 3 National Engineering Laboratory for Big Data System Computing Technology, Shenzhen University, Shenzhen 518060, China; shamsher@szu.edu.cn; 4 School of Computer Science, College of Science, Mathematics and Technology, Wenzhou-Kean University, Wenzhou 325015, China

**Keywords:** intrusion detection system, big data, transfer learning, game theory, network traffic, cybersecurity

## Abstract

Internet of Things (IoT) applications and resources are highly vulnerable to flood attacks, including Distributed Denial of Service (DDoS) attacks. These attacks overwhelm the targeted device with numerous network packets, making its resources inaccessible to authorized users. Such attacks may comprise attack references, attack types, sub-categories, host information, malicious scripts, etc. These details assist security professionals in identifying weaknesses, tailoring defense measures, and responding rapidly to possible threats, thereby improving the overall security posture of IoT devices. Developing an intelligent Intrusion Detection System (IDS) is highly complex due to its numerous network features. This study presents an improved IDS for IoT security that employs multimodal big data representation and transfer learning. First, the Packet Capture (PCAP) files are crawled to retrieve the necessary attacks and bytes. Second, Spark-based big data optimization algorithms handle huge volumes of data. Second, a transfer learning approach such as word2vec retrieves semantically-based observed features. Third, an algorithm is developed to convert network bytes into images, and texture features are extracted by configuring an attention-based Residual Network (ResNet). Finally, the trained text and texture features are combined and used as multimodal features to classify various attacks. The proposed method is thoroughly evaluated on three widely used IoT-based datasets: CIC-IoT 2022, CIC-IoT 2023, and Edge-IIoT. The proposed method achieves excellent classification performance, with an accuracy of 98.2%. In addition, we present a game theory-based process to validate the proposed approach formally.

## 1. Introduction

**Figure 1:**
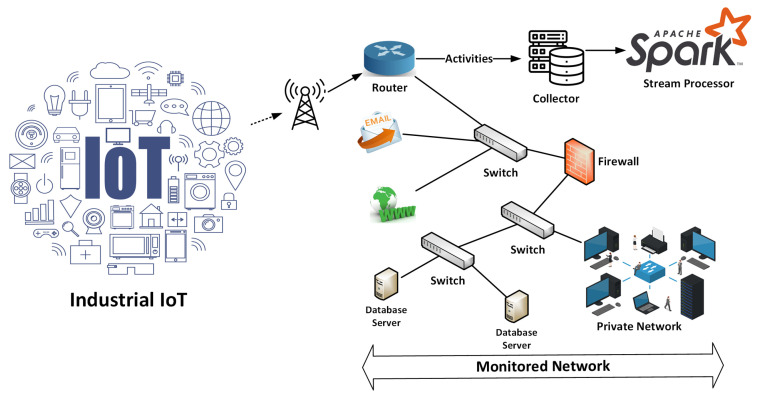
Monitoring of IoT-based network traffic flows using Spark stream processor.

The adoption of Internet of Things (IoT) technologies and systems is accelerating at an unprecedented rate. Modern IoT systems work on a far larger scale than the individual level, with interconnected devices dispersed throughout entire cities or countries. IoT devices can efficiently acquire, send, and process large amounts of data due to increased connectivity speed and bandwidth [1]. Together with their accumulated data, these IoT devices present considerable prospects for developing and delivering smart services in various sectors, including autonomous vehicles, automatic monitoring, and smart cyber-physical networks [2,3]. However, the obtained IoT data may contain personal information, emphasizing the importance of privacy protection and stringent data security procedures. Modern IoT and distributed systems must improve their capacity to effectively identify and thwart network intrusions to resolve the rising issues regarding privacy and security. Techniques for Network Intrusion Detection Systems (NIDS) based on machine learning or deep learning have received much attention in the scientific community [4,5]. Their aim is to mitigate misuse or deviations within IoT infrastructures and systems. While NIDS has proven effective in detecting malicious network activity, a significant limitation is the inability to identify new forms of network intrusions due to the challenges posed by big data processing and related imbalances [6]. Furthermore, existing machine learning algorithms for intrusion detection struggle with multimodal data, emphasizing the significance of further study into hybrid feature engineering. Big data is generated via network traffic, system events, and logs. Cybersecurity is improved by using big data analytics and related approaches to analyze data flows, investigate events, and regularly detect anomalies, shifts, and threats in network traffic, as shown in Figure 1. Remote routers properly collect and track the network traffic that IoT devices generate. These routers send such real-time data to Spark stream processors for efficient processing. The AMP Lab at UC Berkeley developed Spark, a platform for distributed computation [7]. A Spark-based platform can dynamically and efficiently manage massive amounts of generated data. Due to the expansion of large data structures, networking system speeds, and intruder techniques, intelligent intrusion detection remains an essential research challenge. Spark incorporates several integration techniques across multiple big data systems, enabling it to quickly handle enormous amounts of data [8,9]. Its memory-driven Resilient Distributed Dataset (RDD) method is designed for ensembles and recurrent computations and permits intermediary data caching in storage. As a result, the Spark model provides particular benefits for processing massive volumes of data through various optimization approaches, including partitioning, caching, API selection, and more.

Traditional Machine Learning (ML) and Deep Learning (DL) methods in NIDS have prioritized model performance over in-depth network semantic analysis. NIDS evaluates data from hosts and network traffic to detect intrusion attempts. Intruders regularly utilize malicious scripts to disrupt IoT network functionality. Semantic-based feature engineering can be applied to behavioral segmentation to detect potential malicious scripts. This strategy entails evaluating network data semantically to generate training features before using them for classification. Nonetheless, text-based behaviors such as code obfuscation, insertion, and re-ordering present difficulties. In contrast, image-based feature analysis is frequently used, capturing a wide range of structural information such as memory, process, and header details. This enables visual images to retrieve dynamic or obfuscated data of any type [10,11,12]. However, this strategy may disrupt the overall structure of network traffic, making it harder to target specific scripts such as DDoS or authentication protocols. It is also entirely dependent on the properties of images, which leaves it open to attacks that impair image quality. To address these concerns, it is possible to employ multimodal features that combine text and visual features to detect potentially harmful scripts and risky behaviors such as memory or resource usage. This unique multimodal technique accurately identifies a wide range of intrusion threats. This paper introduces an innovative approach for detecting IoT-based NIDS involving analyzing and characterizing network-based multimodal features. A crawler is developed to gather network flows from PCAPs for in-depth study. Subsequently, word2vec is used to extract trained vocabulary features. The network-based byte stream is then converted into an image. We develop a powerful NIDS by integrating text-based and visual features. Our findings show that these two features enhance one another, resulting in a higher intrusion detection rate. The proposed methodology establishes a strong foundation by incorporating anomaly detection and signature-based approaches. Although the current focus is on signature-based detection approaches, including word2vec transfer learning to extract features from harmful scripts, the system can be readily adapted to detect anomalies. This adaptability provides the ability to detect network anomalies. Anomaly detection algorithms can be developed using dynamic feature extraction techniques and enhanced streaming data processing capabilities. This development can enable the IDS to detect abnormal network activity in real-time, thereby improving signature-based detection. Consequently, it can offer a comprehensive defense against emergent threats. The main contributions of this paper are as follows:

- We proposed custom datasets crawled from PCAPs in the CIC-IoT 2022 and 2023 datasets. Numerous attacks are assembled, including camera-based flood, DDoS, RTSP brute force, etc. Furthermore, PCAPs are mined for various camera-based attacks to analyze abnormal visual surveillance behavior.
- The optimization approach, implemented within the Spark framework, efficiently extracts insights from huge datasets. Comparative analysis of different optimization strategies is used to determine how big data behaves and to improve the workings of the NIDS. The word2vec transfer learning approach extracts trained features from dangerous scripts while minimizing data transmission overhead. This strategy uses semantic anchors to focus on specific network attacks involving malicious scripts.
- We design a method that converts the network bytes to an image to analyze the visual features. A malware-to-image conversion algorithm is developed that can transform the byte stream into a grayscale image. The texture features are then extracted from visuals using an attention-based Residual Network (ResNet)-trained model. The text and texture features are then combined for effective IoT-based NIDS.
- A game theory-based method is designed to validate the performance of the proposed method, supporting the use of the Nash equilibrium and mathematical formulations to develop a reliable and trustworthy IoT-based IDS system.

The remainder of the paper is organized as follows: Section 2 describes the relevant and recent literature; Section 3 describes the proposed approach; Section 4 provides an extensive experimental analysis; Section 5 presents the IDS game theory-based study; and Section 6 concludes the work.

## 2. Related Work

Numerous research studies have been proposed on IoT security using IDS customized to the IoT ecosystem’s characteristics. Stephen et al. [13] designed an approach renowned for its mobility, composite structure, and centralized design. This method is intended to detect Hello Flood and Sybil attacks on IoT networks that use the Low Power and Lossy Networks (RPL) protocol for routing. Their method uses detection measures, including the number of packets received and delivered, to verify the intrusion ratio (IR) via the IDS agent. Raza et al. [14] designed the SVELTE method, a real-time IDS for the Internet of Things. It includes a 6LoWPAN Mapper, an intrusion detection module, and a small firewall. The method examines traced data to detect unauthorized network access and has been particularly tested for its capacity to identify faked or changed information, sinkholes, and selective forwarding threats. Shreenivas et al. [15] made SVELTE better by adding an intrusion detection module with an Expected Transmission (ETX) measure to find bad activities happening on the network. They also suggested employing a geographical indicator to identify hostile nodes engaged in ETX-based network attacks. Combining the EXT and rank-based methods revealed a rise in the overall True Positive (TP) rate. Pongle et al. [16] proposed an IDS with centralized and distributed architectures, which they constructed and tested using simulated scenarios and networks. This technique is specifically designed to detect routing attacks such as wormholes. Jun et al. [17] presented an event-processing-based IDS for IoT. For attack detection, this specification-based system employs complicated event-processing algorithms. It collects IoT data, extracts events, and matches them to rule pattern repositories to detect security instances. While it is more efficient than standard IDS, it does place a large demand on the CPU. Summerville et al. [18] designed an IDS for IoT by combining a deep packet inspection approach with a bit-pattern strategy. With feature selection using overlapping n-grams, network payloads are considered as bit-patterns. The bit pattern and n-grams are said to be matched when corresponding bits line up in every location. The false positive rate of the system decreased substantially when it was evaluated via the execution of four assaults [19]. Ioulianou et al. [20] proposed a hybrid lightweight signature-based IDS to counter two DoS attacks: Hello flooding and version number alteration. Despite the excellent findings, it is vital to highlight that their system was tested in a simulated environment using Cooja.

Recent research has shown increasing interest in using big data gathered from network traffic to find different patterns and classify harmful activity. The proliferation of big data has facilitated the advancement of more precise IDS by implementing ML and DL methodologies [21,22]. Gupta et al. [23] used Spark streaming to perform real-time network analysis. Their research revealed several streaming analytics issues, notably spotting port scans, tracking mirrored breaches, and analyzing application efficiency. Using programmed switches such as OpenFlow switches allowed for the collection of 95% of query-related activity. Mustapha et al. [24] used Apache Spark to compare the efficacy of four popular classification methods for detecting intrusions in network traffic: SVM, NB, DT, and RF. The UNSW-NB15 dataset was used to assess detection accuracy and forecast time. Samed et al. [5] developed an IDS using a hybrid deep learning (HDL) network with CNN and LSTM models. To reduce the influence of data imbalance on system performance, they used the Synthetic Minority Oversampling Technique (SMOTE) and Tomek-Links (STL) sampling methods. The PySpark application within Apache Spark showed a remarkable classification accuracy of 99.17%. A statistical IDS was put forth by Kamran et al. [25] to detect fraudulent information attacks and additional forms of intrusion. They investigated the consequences of numerous parameters using simulation and analysis under various traffic situations, presenting their modeling findings graphically and quantitatively. A cooperative information exchange application layer (IDS) was used to compare and evaluate the methodology. The CIDDS-001, CICIDS17, and CICDDoS19 datasets were used by Pontes et al. [26] to design an energy-based method for finding intrusion attacks. Traffic flows were classified as benign or detrimental according to their energy values. To accomplish this, the authors utilized real-world data and executed Mixed Integer Linear Programming (MILP). Their method reduced the Total Cost of Ownership (TCO) by 35% compared to server count and 43% compared to turnaround time.

Business applications frequently produce large amounts of log data to track operational conditions and network utilization metrics. An IDS generally necessitates the examination of PCAP files, which contain unstructured network data. The data may contain vital characteristics of specific attacks, including names, references, and host details. Semantic preprocessing of network data removes noise and extracts relevant information, potentially relieving network-based IDS [27]. Seyyar et al. [28] suggested a web IDS solution that uses network traffic to categorize URLs as benign or malicious. The technique uses the BERT transformer model and CNN to categorize distinct types of attacks during the URL analysis phase. Li et al. [29] used the word2vec model and weighted mapping of HTTP data to design a low-dimensional feature vector for semantic extraction. The detection accuracy, true positive rate, and false positive rate were high on datasets such as HTTP CSIC 2010, UNSW-NB15, and Malicious URLs. Min et al. [30] advocated a statistical and payload-based IDS strategy using word semantics and text-CNN to extract relevant attributes from payloads.

The work described in this paper overcomes several deficiencies in existing intrusion detection systems. Such difficulties include scalability issues with huge datasets, which create processing delays and limited feature representation based only on text. Other limitations include insufficient threat detection due to a lack of connectivity between text and image data, difficulty identifying complex assaults in diverse IoT environments, and reliance on manual rules, limiting adaptability. The suggested solution addresses these issues by combining text and picture data. In addition, transfer learning improves accuracy and adaptability in IoT security and optimizes algorithms for greater efficiency. This integrated strategy improves the system’s ability to respond quickly to emerging threats while capturing a wider range of attack patterns, ultimately increasing overall efficacy.

## 3. Proposed Method

**Figure 2:**
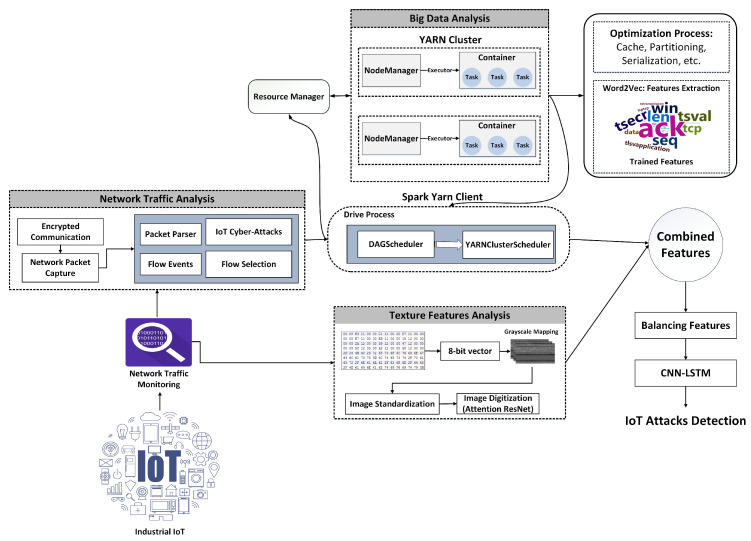
Proposed framework for enhancing IoT security using multimodal data representation and big data.

Figure 2 depicts the overall process of extracting multimodal features and detecting attacks. We use multimodal features combining text and visual elements to detect malicious attacks. Textual features are collected from network traffic using Spark-based optimization algorithms and a transfer learning approach. The network bytes are transformed into images to extract textural information. An algorithm is created to handle this conversion, and texture features are extracted by tuning the attention-based ResNet. The trained text and texture features are integrated and used as multimodal features to classify different attacks. These novel multimodal features correctly identify a wide range of intrusion threats.

### 3.1. Network Data Preprocessing

The PCAP includes the communication logs utilized by IoT devices. Each message is preserved in an unreadable format. By filtering out essential network-related activities (e.g., HTTP, TCP, DNS), Wireshark is employed to analyze the PCAP file. Such flow activities could include various data, including host data, protocol, both the sending and receiving IP addresses, and the time. Analyzing these transit events makes distinguishing between harmless and malicious network behavior possible, as shown in Algorithm 1. An effective IDS is developed by combining this information with a big data platform that employs transfer learning. Nevertheless, this type of data is perturbed and might not possess significant interpretations that could aid in detecting diverse attacks. As a result, all chaotic flow events are eliminated from this data. We accomplished this by developing a semantic crawler that processes all flow activities and transforms them into substantial trends. The subsequent steps provide additional illustrations of the crawling procedure:

- To avoid redundancy, eliminate repetitive features in sequence within input sets.
- The dataset excludes brief sequences that lack sufficient data to determine relevant network behavior.
- Sequence length homogeneity is critical for effective IDS, as varying lengths might confuse neural network models. To achieve balance, this technique employs a predetermined sequence length, designated as L. Patterns longer than L retain their initial L elements, while shorter patterns are equalized using zero-padding. The complete architecture is shown in Figure 2.

**Algorithm 1:** Texture features  **Input**: Network Traffic  **Output**: Texture feature  initialization
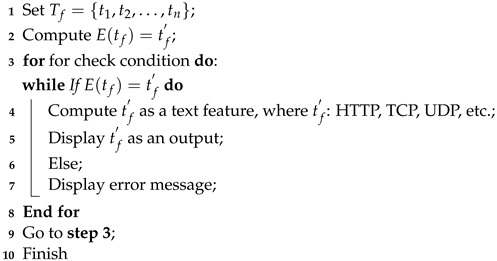


### 3.2. Texture Features Analysis

**Figure 3:**
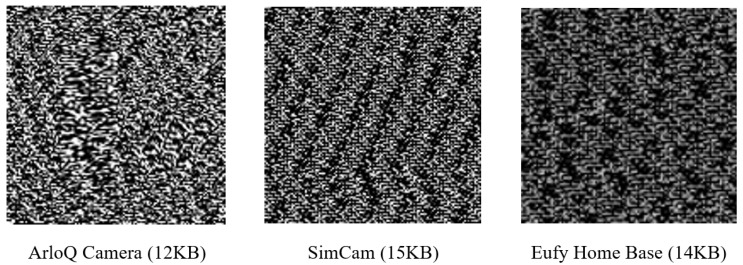
A chunk of grayscale images (128 × 128) extracted from network traffic.

Texture attributes analyze intrusions as harmful activities that change to elude static and dynamic detection methods. This novel method extracts textural information from bytes as images, eliminating the requirement for intrusion fingerprints or reverse engineering, as shown in Algorithm 2. Our approach includes a bytes-to-image conversion method that creates grayscale images from network traffic. The packet data are initially parsed to extract bytes from PCAPs by retrieving relevant byte streams from network packets. These byte sequences are subsequently converted into a string of unsigned 8-bit integers in the form of images. The retrieved images are standardized to 128 × 128 pixels in size to facilitate effective processing. This method is distinguished by its ability to effectively reduce the size of large PCAP files. For example, megabytes of PCAP data can be reduced to smaller, more standardized images, as shown in Figure 3. The versatility of image-based IDS makes them ideal for capturing a wide range of structural data, including storage, processes, headers, etc. By utilizing these visuals, advanced image processing and machine learning techniques such as deep learning models can be employed to identify patterns and anomalies that conventional methods may have overlooked. This increases the effectiveness of the proposed IDS.
**Algorithm 2:** Texture features in bytes  **Input**: Network Traffic in bytes  **Output**: Texture feature in bytes  initialization
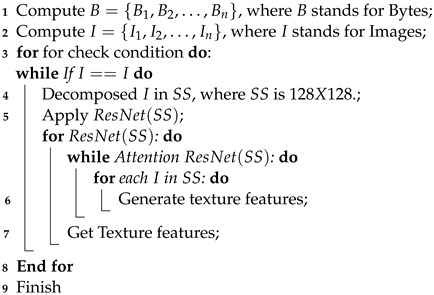


**Figure 4:**
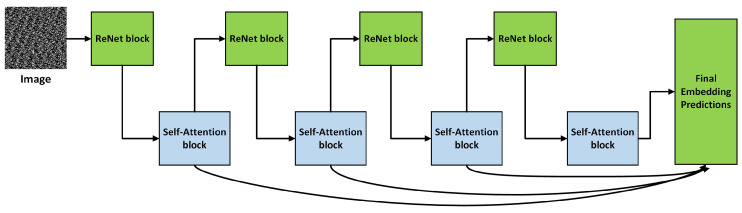
Attention-based ResNet for texture features extraction.

In neural networks, attention frequently refers to strategies that allow the model to focus on specific portions of the input sequence or image while making predictions. A Residual Network (ResNet) is a deep neural network that uses residual blocks or skip connections to address the vanishing gradient issue [31]. Figure 4 shows how we constructed the attention-based ResNet to extract image texture information. This network’s attention-aware characteristics are produced via several attention modules. As the depth of the layers grows, these attention-aware characteristics evolve gradually throughout modules. The extracted images are used as input to the ResNet blocks. The details are as follows:

(a) **Hierarchical network composition**: Several attention modules are assembled in a layered framework to develop residual attention networks. This stacking structure is the basic implementation of the mixed attention mechanism, which allows for integrating multiple types of attention into distinct modules.
(b) **Attention Residual Learning**: A significant performance drop would be the outcome of directly stacking the attention modules; to address this, we present a method for learning attention residuals that can enhance the performance of a residual attention network with several layers.

Adding self-attention to the ResNet increases its ability to store long-term dependencies, assess global context, and focus on key features. This combination improves data transmission, enables feature learning at several sizes, and works across multiple tasks, making it an effective solution for various applications.

### 3.3. Transfer Learning

We use a pre-trained word2vec model as a transfer learning approach to extract semantic features from large network data. The neural network uses vector features to detect various types of attacks. A fixed-size feature vector, denoted as L, is obtained following a network traffic analysis. Although one-hot encoding can handle these characteristics, it is unsuitable for huge datasets. The model is initially trained on a large corpus of text related to network traffic analysis. During training, the model learns to generate dense vector representations (word embeddings) for each corpus word, capturing semantic interpretations and additional context. To optimize the model parameters, including neural network weights, gradient descent minimizes a loss function that compares expected and actual outputs. This iterative optimization reduces the gap between anticipated and observed outcomes, improving the model’s performance with each iteration. Then, transfer learning is used to apply previously acquired information to detect cyberattacks in IoT network traffic. Word2vec transfer learning gradually improves pre-trained word embeddings by leveraging IoT network traffic data. This continual fine-tuning refines the embeddings, improving their ability to capture the contextual relevance of network traffic features. In this way, vector semantics are improved by establishing geometric connections between them. Word2vec trains and extracts features using dynamic fine-tuning. This produces numerous vectors with comparable meanings for each feature, allowing for multiple interpretations of the same feature. The model can better categorize and mitigate threats in various network scenarios by exploiting the semantic understanding of data acquired by the original model through transfer learning.

The Spark platform task and the word2vec transfer learning algorithm make it easier to obtain trained features from network traffic, as shown in Algorithm 3 [32]. The org.apache.spark.ml.feature class in Spark is responsible for implementing dynamic word2vec. This class uses distributed computation capabilities to simplify vector generation from large text corpora and accelerate word2vec training. Converting the data into the Data Frame format before beginning the training process with the word2vec Spark class is essential. Subsequently, the fit function must be invoked. After training, we use the Discover Synonyms method to find phrases similar to a particular word. Processing speeds for Spark and Gensim may differ due to their respective algorithms and implementation techniques. Word2vec, which uses distributed processing capabilities, can outperform Gensim when dealing with large datasets. When working with small datasets, Spark may perform slower than Gensim due to data translation to the Data Frame format and the execution of multiple I/O operations.
**Algorithm 3:** Trained crawling texture features  **Input**: $\left\{\theta_{t},\theta_{i}\right\}$, where $\theta_{t}$ is for text feature and $\theta_{i}$ for texture feature  **Output**: $\theta_{f}^{\prime }$ IoT-based IDS classification
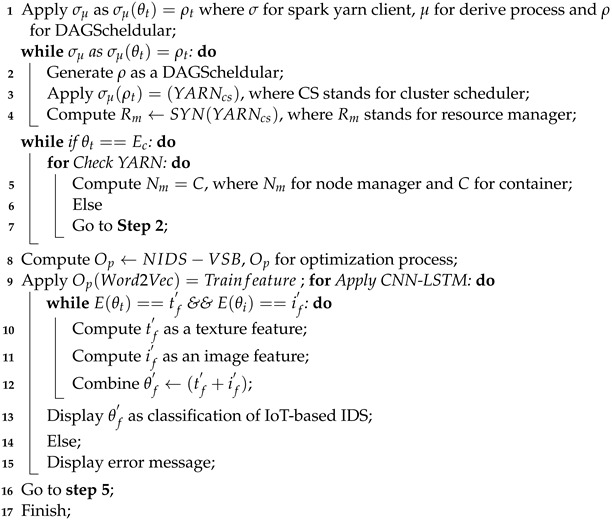


### 3.4. Big Data Analysis

We investigated several optimization methods to analyze in-depth analysis for computation speed when dealing with large datasets, including partitioning, caching, serialization, data storage, and API selection [33].

(a) **Partitioning**:The number of partitions is an important parameter that significantly impacts how efficiently Spark analyzes data. When the partition count is limited, computational resource utilization on individual nodes may be insufficient. On the other hand, too many partitions may increase network transmission and node scheduling costs, limiting processing efficiency. The unique processing and storage capacities of each node in a distributed computing system make it advantageous to synchronize the number of Spark partitions with the number of nodes. We aimed to select a partition count equal to the number of nodes that allows for the most efficient use of resources on each node. This alignment enhances data processing efficiency by reducing irrelevant network traffic and node scheduling overhead.
(b) **Caching**: Spark is configured to cache data in memory, although disk storage is also an option. The cache can be set to employ disk storage to achieve the desired results. Spark might theoretically achieve faster processing times by caching data in memory, which has faster read-write and response times than disks. Besides improving overall data processing performance, this strategy lowers data access costs by reducing the frequency of disk read-write operations. However, disc caching has the advantage of a longer data retention period and greater storage capacity. On the other hand, its response time and read-write speed are quite slow, limiting data processing efficacy. Disk caching is often more appropriate in circumstances requiring significant data storage capacity without overwhelmingly emphasizing frequent access and computation. It is critical to note that storing data in memory can result in insufficient memory or out-of-memory (OOM) issues when memory space is limited or the dataset is very large. In such instances, using disk caching or studying other optimization methods to improve data processing throughput becomes crucial.
(c) **Serialization**: Spark utilizes the Kryo and Java serialization mechanisms. Despite the widespread use of Java serialization, the default application of this strategy is inefficient, leading to large amounts of serialized data, expensive disk storage, and network transmission expenses. In contrast, Kryo serialization provides an efficient binary alternative. In addition to reducing the quantity of data, it speeds up the serialization and deserialization procedures and lowers disk storage and network transmission costs. As a result, Kryo serialization is suggested in scenarios requiring high performance.
(d) **Data storage**: Spark supports various formats, including CSV, JSON, XML, PARQUET, ORC, AVRO, and others. Choosing parquet files with fast compression can improve Spark jobs, resulting in better performance. Parquet files internal to Spark contain metadata, including schemas, data types, and additional pertinent information. As a result, implementing parquet files can significantly accelerate processing and improve the overall efficacy of information management.
(e) **API selection**: The three types of Spark APIs are DataFrame, DataSet, and RDD. RDD is used in reduced-level processes, which have a restricted number of optimization strategies. DataFrame is generally the best option, as it employs the catalyst optimizer, which produces a query plan that enhances performance. DataFrame also has minimal trash collection overhead. DataSets offers high-type safety during serialization by integrating the encoder and using Tungsten as a binary serializer. The amount of data and the techniques employed influence how rapidly RDD, DataFrame, and DataSet in Spark process information. DataFrame and DataSet frequently beat RDD by employing Spark SQL’s optimizer and code generator. In addition to binary serialization and deserialization, they provide columnar storage. Furthermore, both DataFrame and DataSet enable rigorous typing, which allows for the examination and avoidance of type problems during compilation. DataSet requires more code and type declarations than DataFrame but has faster processing as it employs the Tungsten engine, a high-performance in-memory management and serialization engine. As a result, the right API must be chosen based on specific parameters, such as data types and processing needs.

The complexity of high-dimensional network traffic data makes it difficult to use big data for intelligent IDS effectively. Selecting useful characteristics while lowering dimensionality remains a challenge. Spark’s DataFrame, memory caching, Kryo serialization, PARQUET file storage, and adjustable partition numbers based on node count dramatically improve processing performance and data efficiency, especially with huge datasets. Several important features of the IDS make proactive threat identification and response possible in real-time. Utilizing optimization strategies within the Spark framework is one of these methods for efficiently processing streaming data. Furthermore, the word2vec transfer learning approach enables real-time adaptive feature extraction from harmful scripts. The system can rapidly identify potential risks by analyzing irregular visual surveillance behavior by scanning PCAPs for camera-based assaults. The IDS is ensured to be adaptable and resilient in dynamic environments by utilizing a formal validation method based on game theory. This method provides an exhaustive framework for assessing the effectiveness of proactive strategies.

### 3.5. Deep Learning: CNN-LSTM

**Figure 5:**
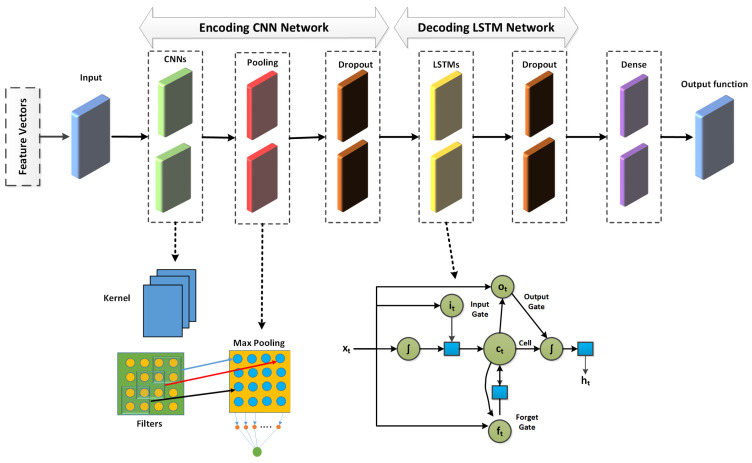
Deep CNN-LSTM architecture for IoT-based IDS.

The proposed method leverages a CNN-LSTM [34] framework to detect intrusions in network systems immediately using the benefits of both models. Within CNN networks, max-pooling layers extract features for the next fully connected layer. On the other hand, the suggested CNN-LSTM model bypasses the fully connected layer in favor of the LSTM layer for processing deep features. The LSTM network detects long short-term correlations, whereas the CNN network is good at representing and perceiving network feature vectors. Figure 5 depicts the two steps of the defined CNN-LSTM model. The first phase involves implementing convolution, dropout, and max-pooling layers, whereas LSTM and dropout layers are utilized in the second phase. Convolution layers encode the network feature set and LSTM layers decode it. Data are flattened before being transmitted to a completely connected layer to optimize the performance of the IDS. LSTMs depend on the fundamental elements comprising the cell state and its gates. The cell state acts as a data connection route, transporting relevant features across the sequence-aligning system. The memory module of the LSTM is essential; it consists of one storage unit and three interaction gates (input, forget, and output). When data are communicated using the sigmoid activation function, values close to 0 indicate forgetting, and values close to 1 indicate retention. The input gate decides what information to keep at a time “t”, the forget gate controls data flow at a time “t minus 1”, and the output gate manages parameter settings and oversees hidden state actions. The sigmoid and tanh functions are used to integrate the past and present input and calculate the hidden state, which predicts the hidden state information that will be revealed later. Equations (1)–(6) describe how the model works.

(1) $$i_{t}=\sigma \left(V_{ixt}+W_{i}h_{\left(t-1\right)}+b_{i}\right)$$

(2) $$f_{t}=\sigma \left(V_{f}xt+W_{f}h_{\left(t-1\right)}+b_{f}\right)$$

(3) $$\hat{c_{t}}=tanh\left(V_{c}X_{t}+W_{c}h_{\left(t-1\right)}+b_{c}\right)$$

(4) $$c_{t}=\left(f_{t}AC_{t}+W_{c}h_{\left(t-1\right)}+i_{t}A\hat{c_{t}}\right)$$

(5) $$o_{t}=\sigma \left(V_{o}x_{t}+W_{o}h_{\left(t-1\right)}+b_{o}\right)$$

(6) $$h_{t}=o_{t}Atanh\left(c_{t}\right).$$

The symbol $x_{t}$ represents the input at time “t”, the symbols $x_{*}$ and $w_{*}$ represent the weight matrices, the symbols b and h represent the bias and hidden states, respectively, the symbols σ and tanh represent the activation functions, and the letters $i_{t}$, $f_{t}$, $o_{t}$, and $c_{t}$, represent the input gate, forget gate, output gate, and memory cell, respectively.

Scalability is critical to our system’s response to the increasing hazard of flood attacks on IoT applications. By integrating multimodal big data representation with transfer learning, our system can dynamically adapt to various attacks. Spark-based optimization ensures that huge datasets are processed smoothly. We use transfer learning to extract semantically rich features, improving adaptation to evolving threats. Furthermore, we employ innovative approaches such as transforming network data into images and applying ResNet to extract texture properties. This combination makes it possible to classify attacks accurately. To demonstrate scalability, we extensively tested several typical IoT datasets, such as the CIC-IoT 2022 and 2023 datasets. In addition, by including validation based on game theory, we ensure that our system is resilient and scalable, making it capable of efficiently handling future issues.

## 4. Experimental Results

### 4.1. Datasets

We used three standard datasets to investigate the efficiency of the proposed method, namely, CIC-IoT 2022 [35], CIC-IoT 2023 [36], and Edge-IIoTset [37]. Canadian Institute for Cybersecurity datasets were used in these experiments. IoT devices were set up and used to examine network traffic behavior. The CIC-IoT 2022 dataset is generated using Wireshark and dumpcap in six distinct types of experiments. Dumpcap is used for semi-automated tests, whereas Wireshark is utilized for hands-on testing. The tests are divided into six categories: power, idle states, interactions, scenarios, active states, and attacks. For this investigation, we collected network flows associated with eleven different types of flood attacks, including Amcrest, Arlo Basestation Camera, ArloQ Camera, Borun Camera, DLink Camera, HeimVision Camera, Home Eye Camera, Luohe Camera, Nest Camera, Netatmo Camera, and SimCam, during device activation and interaction. The CIC-IoT 2023 dataset operates in real-time and serves as a benchmark and dataset specifically selected for evaluating extensive attacks in the IoT domain. Its main goal is to introduce a unique and comprehensive dataset on IoT threats for use in developing security analytics applications in real IoT operations. To this end, it implements 33 attacks on 105 IoT devices. The attacks are classified into seven types: DDoS, DoS, Recon, Web-based, Brute Force, Spoofing, and Mirai. We organized ten unique DDoS attacks to conduct the experiment: SYN_Flood, TCP_Flood, SynonymousIP_Flood, UDP_Flood, ICMP_Flood, PSHACK_Flood, RSTFINFlood, HTTP_Flood, ACK_Fragmentation, and ICMP_Fragmentation. Finally, the Edge-IIoTset dataset contains data from more than ten IoT devices, including inexpensive digital temperature and humidity sensors. It includes 14 DoS/DDoS, information gathering, man-in-the-middle, injection, and malware attacks on IoT and IIoT communication protocols. The dataset comprises alarms, system resources, logs, and network traffic, with 61 significantly related characteristics out of 1176 total features.

### 4.2. Performance Measures

To comprehensively analyze the proposed approach, we used a set of five distinct assessment metrics: precision, recall, f1-score, accuracy, and the confusion matrix. True Positives (TPs) and True Negatives (TNs) are accurately classified based on how much of each type of network traffic is normal and abnormal. In contrast, a substantial fraction of regular and aberrant network traffic is incorrectly labeled as False Positive (FPs) and False Negative (FNs). The overall classification efficacy was assessed by calculating the accuracy, which is determined by dividing the number of correctly classified instances by the total number of instances. The evaluation matrices are shown in Equations (7)–(10).

(7) $$\mathrm{Recall}=\frac{\mathrm{FP}}{(\mathrm{FP}\;+\;\mathrm{TN})}$$

(8) $$\mathrm{Precision}=\frac{\mathrm{TP}}{(\mathrm{TP}\;+\;\mathrm{FP})}$$

(9) $$F1-\mathrm{score}=\frac{(2\;*\;\mathrm{TP})}{(2\mathrm{TP}\;+\;\mathrm{FP}\;+\;\mathrm{FN})}$$

(10) $$\mathrm{Accuracy}=\frac{(\mathrm{TP}\;+\;\mathrm{TN})}{(\mathrm{TP}\;+\;\mathrm{TN}\;+\;\mathrm{FP}\;+\;\mathrm{FN})}$$

### 4.3. Results Analysis

**Figure 6:**
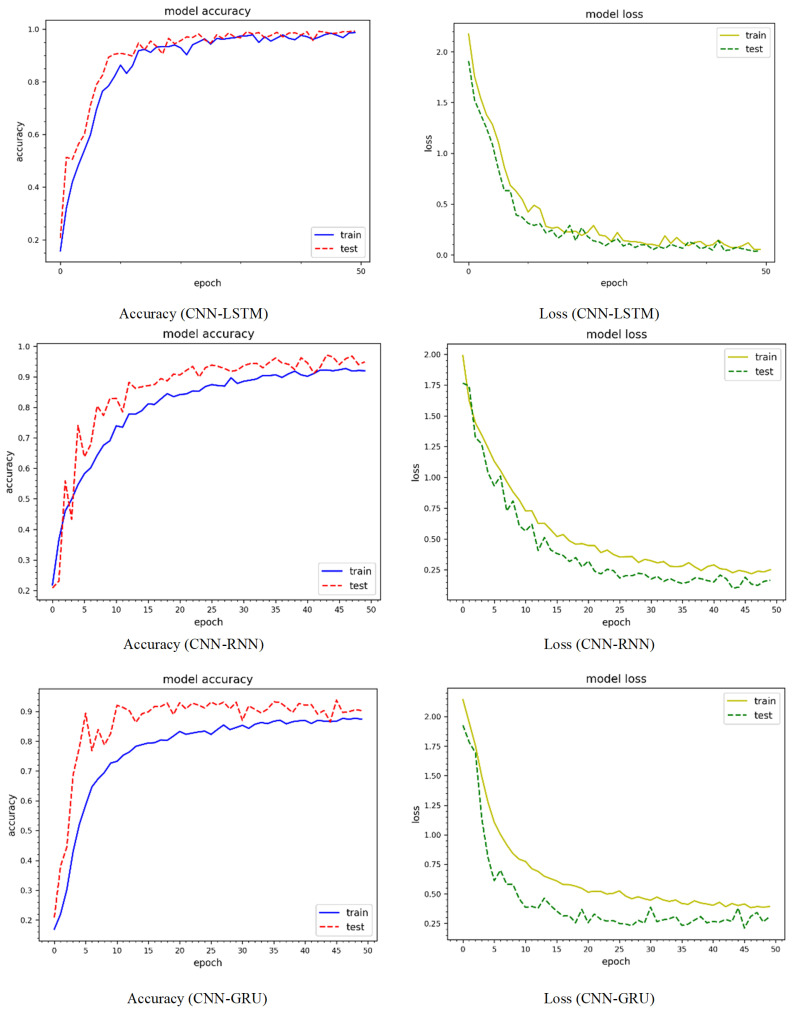
Accuracy and loss dynamic curves on the CIC-IoT 2022 dataset.

**Figure 7:**
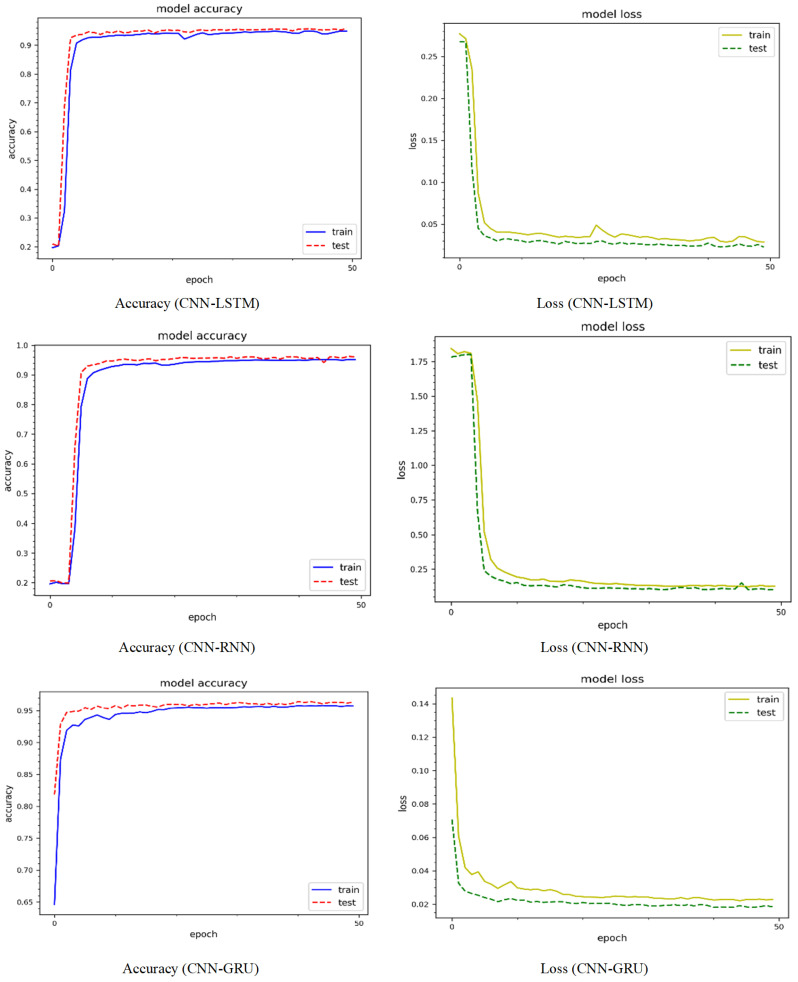
Accuracy and loss dynamic curves on the CIC-IoT 2023 dataset.

Figure 6 shows the epoch curves for training and testing data obtained from the CIC-IoT 2022 dataset. To compare the performance of the proposed method, we used three prominent deep learning models: CNN-LSTM, CNN-RNN, and CNN-GRU. The blue color represents training data, whereas the red color represents testing data for model accuracy. Similarly, yellow represents the training data, whereas green represents the testing data for model loss. Using CNN-LSTM, the model accuracy curves comprising training and testing show behavior between 10% and 99%. Both curves behave similarly, with minor variations at different epochs. For instance, there is an abrupt decrease in the testing curve of up to 85% on the 22nd epoch and 83% on the 24th epoch. When compared to the accuracy curves, the loss curves react differently. For instance, the training and loss values are initially large, but drop steadily after a few epochs, finally yielding a minimum loss of approximately 3%. The training and testing curves for the CNN-RNN model accuracy range between 22% and 95%. Similarly, for both the training and testing curves the minimum model loss is around 10%. The model accuracy and testing curves for the CNN-GRU model range from 19% to 90%. Both curves exhibit significant variability, particularly for the testing data. The model loss is roughly 17% for both the training and testing curves. The CNN-LSTM model performs better, whereas the CNN-GRU model performs worse. Figure 7 shows the accuracy and loss curves of the model using the CIC-IoT 2023 dataset. The model accuracy curves for the CNN-LSTM model, including training and testing, range from 19% to 96%, while the minimum loss is around 4%. The CNN-RNN model accuracy curves range from 21% to 96%, with a minimum loss of around 8%. Similarly, for the CNN-GRU model, the accuracy curves range from 5% to 95%, with the testing curve beginning at 82%. Thus, the CNN-LSTM performs better on the CIC-IoT 2023 dataset.

The CNN-LSTM model’s performance metrics on the CIC-IoT 2022 dataset are shown in Table 1. The precision, recall, and f1-score metrics were used to examine the camera-based flood attacks. The Amcrest has a 98% f1-score, 100% recall, and 95% precision, respectively. Comparably, the Arlo Basestation Camera offers 100% f1-score, 99% recall, and 100% precision. For precision, recall, and f1-score, ArloQ, DLink, Hiem Vision, and SimCam all have 100% performance. Overall, the values range from 95% to 100% for all types of flood attacks. The performance metrics for the CNN-RNN model on the CIC-IoT 2022 dataset are shown in Table 2. The performance of this method is marginally less than that of CNN-LSTM. For instance, all sorts of flood attacks have values of 58% to 100% according to the performance metrics. The Luohe Camera shows the lowest performance, with 58% precision, 100% recall, and 74% f1-score. The CNN-GRU model’s performance metrics for the CIC-IoT 2022 dataset are shown in Table 3. In comparison to the CNN-LSTM and CNN-GRU models, it offers the lowest performance metrics. The Luohe Camera has the lowest performance, with 55% precision, 98% recall, and 71% f1-score.

Table 4 displays the performance metrics obtained with the CNN-LSTM model on the CIC-IoT 2023 dataset. A total of ten DDoS attacks were examined. SYN_Flood has a precision of 75%, a recall of 97%, and an f1-score of 85%. Similarly, TCP_Flood has 100% performance across all three performance metrics. SynonymousIP_Flood has the lowest precision (96%), recall (69%), and f1-score (80%). Overall, the performance metrics range from 69% to 100%. Table 5 shows the performance metrics obtained by the CNN-RNN model on the CIC-IoT 2023 dataset. SynonymousIP_Flood has the worst performance, with a precision of 96%, recall of 64%, and f1-score of 77%. SYN_Flood comes second with a precision of 73%, recall of 98%, and f1-score of 83%. Overall, the CNN-RNN model delivers performance metrics ranging from 64% to 100% for all forms of DDoS attacks. Table 6 compares the performance of the CNN-LSTM, CNN-RNN, and CNN-GRU models for average values of the performance indicators. This study was based on both two datasets. The two datasets demonstrate that the CNN-LSTM model delivers the best performance for detecting various intrusions. For instance, on the CIC-IoT 2022 dataset, the CNN-LSTM model has an average precision of 98.1%, recall of 98.4%, f1-score of 97.9%, and classification accuracy of 98.2%. The CNN-LSTM model achieves a 96.4% classification accuracy, 97% precision, 96.1% recall, and 96.1% f1-score on the CIC-IoT 2023 dataset. These results demonstrate that the CNN-LSTM model has the highest performance, whereas the CNN-GRU model has the lowest and the performance of the CNN-RNN model lies in the middle. Table 6 shows the classification performance of the CNN-LSTM model on the Edge-IIoT dataset, which includes 14 different IoT attacks. XSS, Ransomware, and OS Fingerprinting have the highest classification, while MITM has the lowest. Considering the variety of attacks in this dataset, the overall classification performance is impressive at 96.1% for 14 classes. Table 7 shows the performance comparison for the three datasets.

**Figure 8:**
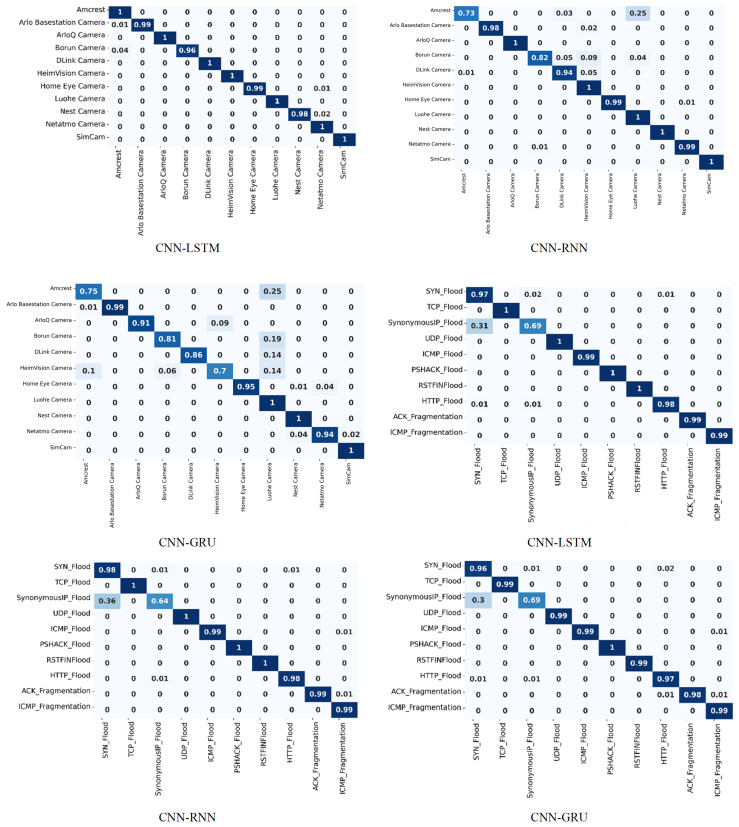
Confusion matrices for the CIC-IoT 2022 and CIC-IoT 2023 datasets.

The confusion matrices for the three deep learning models using both datasets are shown in Figure 8. These matrices examine the correct and incorrect classifications at the class level. This evaluation can comprehensively evaluate how well our suggested approach worked for the specified class. The diagonal values represent the correct classification, while the off-diagonal values represent the incorrect classification. The CIC-IoT 2022 dataset contains eleven types of attacks, whereas the CIC-IoT 2023 dataset contains ten. The CNN-LSTM model performs well on the CIC-IoT 2022 dataset; most classes have 100% classification, such as Amcrest, ArloQ Camera, DLink Camera, etc. However, a few classes exhibit slight misclassifications; for example, the Arlo Basestation Camera has 1% incorrect classification, while the Borun Camera has 4%. Amcrest has the lowest performance when using the CNN-RNN model, with 73% correctly classified and 27% incorrectly classified. Using the CNN-GRU model, the lowest performance for the HeimVision Camera is 70% correct classification and 30% error. The CNN-LSTM model delivers the best classification results for all sorts of attacks on the CIC-IoT 2022 dataset. However, CNN-LSTM delivers the lowest classification on the CIC-IoT 2023 dataset for SynonymousIP_Flood, with 69% correct and 31% incorrect, while the majority have 100% or more than 97% correct classification. The CNN-RNN model’s classification is the next best, while the CNN-GRU model’s classification is the worst.

## 5. Game-Theoretical Perspective on IDS for IoT

Game theory and the development of IDS for the IoT have emerged as a significant study area in recent years. This context has offered insight into cutting-edge approaches for addressing the complex security issues linked to IoT systems. Game-theoretical analysis can help to evaluate and improve NIDS security solutions [38]. While the primary purpose of a NIDS is to detect malicious behavior by monitoring traffic streams, signatures, and anomalies, adding game theory can provide significant strategic insight. This technique contributes to a better understanding of how attackers interact with and respond to security mechanisms [39,40]. This methodology helps to identify optimal processes, analyze equilibrium conditions, and allocate resources more efficiently. Analyzing dynamic and sequential games allows defenders to anticipate attackers’ plans while designing adaptable and cost-effective responses. Incorporating game theory into NIDS increases risk detection, resulting in more innovative and reliable security solutions. Furthermore, researchers have used the theoretical framework of the Nash equilibrium [41] to identify optimal techniques for distributing resources in intrusion detection [42,43]. In addition, evolutionary game theory has been used to analyze the concurrent development of methods employed by attackers and defenders in IoT systems [44,45]. These significant findings emphasize the significance of game theory as a vital tool in advancing IoT-based IDS. Such studies provide new perspectives on security analysis, strategy optimization, and decision-making in a constantly changing field of cyber threats [46,47].

### 5.1. Modeling Game Theory-Based IDS for IoT Security

**Figure 9:**
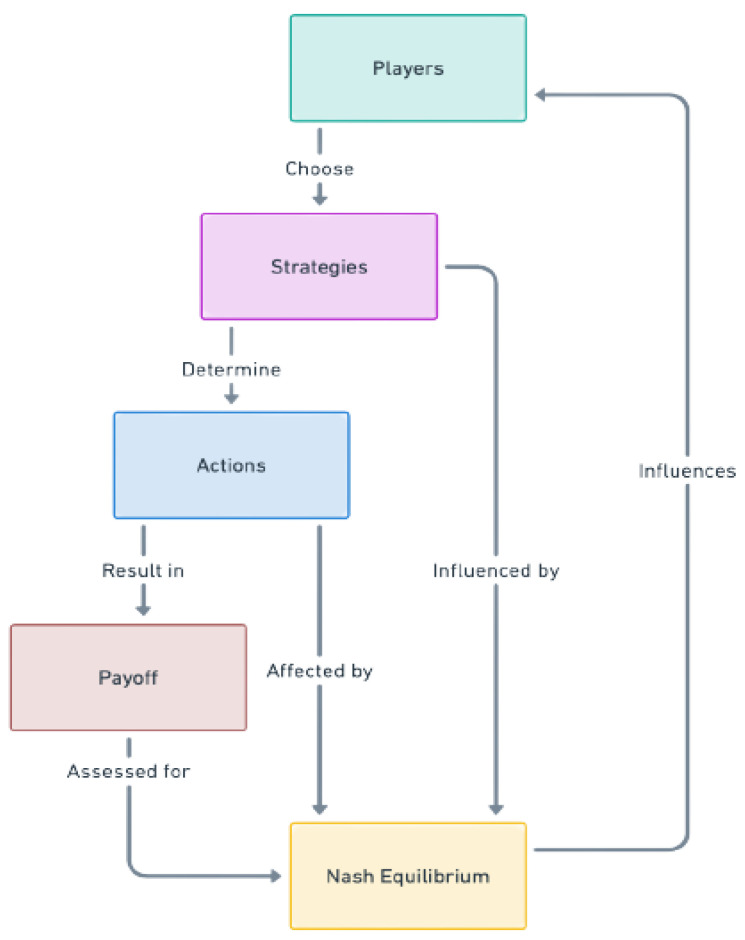
A representation of the cyclic effect of players, strategies, actions, and payoffs on the Nash equilibrium.

Figure 9 presents a complex framework of game theory interactions and recurrent decision-making patterns. It illustrates how players’ strategic choices and actions lead to outcomes that are meticulously assessed to identify the Nash equilibrium (NE) and influence future strategic decisions. The suggested IDS game for the IoT approaches **NE** when both the attacker and the defender cannot boost their payoffs by changing their techniques. At the same time, the other side’s strategy remains unchanged. This balancing demonstrates the resilience and effectiveness of various intrusion detection methods against different attack strategies.

The components of the proposed game-theoretic IDS for IoT security model can be defined as follows:

1. In the context of IoT security, the set of players, denoted as $P=\{P_{1},P_{2},\cdots ,P_{m}\}$ (m≥2) in this game-theoretic model, consists of defenders and attackers. In IoT systems, defenders aim to prevent unauthorized access and protect system stability. At the same time, attackers, driven by objectives such as data pilferage or service disruption, persistently threaten these networks.
2. The concept of ‘activity’ is essential in the game-theoretical analysis of IDS, as it encompasses the strategic decisions made by players, whether attackers or defenders, to optimize their profits. The game’s rules lie in the players’ ability to decide between interacting or dodging binary options. Attackers need to decide whether or not to initiate attacks; at the same time, defenders must choose whether to execute their strategy or adopt a passive approach. This strategic duality defines the game’s interactive essence. Within a formal setting, we shall label P to indicate a player with a collection of non-trivial feasible options. The set A is defined as $A=\{A_{1},A_{2},\ldots ,A_{m}\}$, where every component $P_{n}$ represents a distinct action that the player can execute. The intrinsic depth of a set A contributes to the game’s strategic complexity by offering player P a multitude of possibilities to achieve the most advantageous outcome.
3. In an academic context, a collection of strategies in game theory is represented as $S=\{S_{1},S_{2},\ldots ,S_{m}\}$. These methods include a sequence of planned actions formulated by subjects while considering previous outcomes and their rewards. The efficacy of the attacker’s strategy relies on evading detection while transmitting malicious data packets. On the other hand, the defender’s strategy focuses on identifying unusual changes in entropy and efficiently coordinating defensive actions while minimizing energy consumption.
4. The payoffs are decided according to each participant’s chosen strategies. The collection of payoffs consisting of *m* payoffs for matching strategies can be denoted as $Y=\{Y_{1},Y_{2},\ldots ,Y_{m}\}$. The payoffs are determined by considering the advantages of a successful defense or attack, the cost associated with deploying defensive methods, and the costs borne by attackers. The provided evaluation approach clearly outlines the effectiveness of each defensive strategy against each attack strategy, indicating how well each defense can neutralize a certain sort of attack.
5. Upon analyzing the methods used by attackers and defenders and the resulting benefits, it is crucial to understand how these benefits interact with the strategic structure of the game. Such interactions can be defined by the concept of the Nash Equilibrium (**NE**) [41]. The **NE** is important in game theory, as it represents a scenario in a non-cooperative game where no player can improve their result by independently altering their strategy given that the other players’ strategies remain unchanged. The mathematical representation of the NE is as follows:
  - Consider a game with m≥2 players, denoted as P, each possessing a distinct set of tactics, say, $S_{P}$. Let $s_{r}$ denote the strategy selected by player $P_{r}$ and let $s_{-r}$ indicate the strategies chosen by the remaining players. The utility function for player $P_{r}$ is represented as $U_{P}\left(s_{r},s_{-r}\right)$.
  - A Nash Equilibrium is defined as a strategy profile $\left(s_{1}^{*},s_{2}^{*},\ldots ,s_{m}^{*}\right)$ in which the condition stated below is satisfied for every player $P_{r}$:
    (11) $$U_{r}\left(s_{r}^{*},s_{-r}^{*}\right)\geq U_{r}\left(s_{r},s_{-r}^{*}\right),\;\;\forall s_{r}\in S_{P}.$$

### 5.2. An Analytical Approach for Finding the **NE**

**Figure 10:**
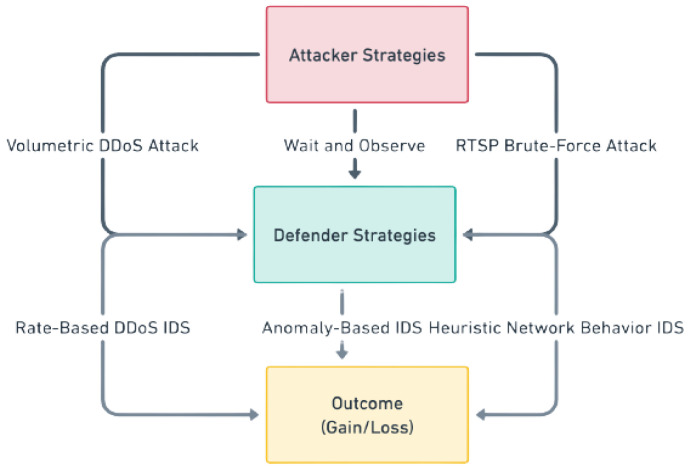
A representation of the proposed strategies of the defender and attacker.

Here, to provide a mathematical perspective on the proposed IDS game-theoretical model, we consider the following three strategies of defenders and attackers (see Figure 10).

The representation of the strategy space for both the defender $\left(S_{D}\right)$ and attacker $\left(S_{A}\right)$ can be identified by the rows and columns in a 3×3 matrix M, as follows.

$$M=\left[\begin{array}{ccc} q_{11} & q_{12} & q_{13}\\ q_{21} & q_{22} & q_{23}\\ q_{31} & q_{32} & q_{33} \end{array}\right]=\left[\begin{array}{ccc} \left(S_{D_{1}},S_{A_{1}}\right) & \left(S_{D_{1}},S_{A_{2}}\right) & \left(S_{D_{1}},S_{A_{3}}\right)\\ \left(S_{D_{2}},S_{A_{1}}\right) & \left(S_{D_{2}},S_{A_{2}}\right) & \left(S_{D_{2}},S_{A_{3}}\right)\\ \left(S_{D_{3}},S_{A_{1}}\right) & \left(S_{D_{3}},S_{A_{2}}\right) & \left(S_{D_{3}},S_{A_{3}}\right) \end{array}\right]$$

In the above matrix M, the element $q_{11}$ denotes the rate-based detection of a DDoS attack by an IDS against volumetric DDoS attacks executed by the attacker. This particular situation entails the utilization of volumetric DDoS attacks by the attacker, which is already known to the defender. Simultaneously, the defense employs rate-based DDoS monitoring to identify and detect attacks efficiently. The calculation of the IDS reward depends on the equilibrium between energy use and the benefits obtained from efficient monitoring, as defined in the following equation:

(12) $$U_{11}\left(D\right)=G_{SD}\left(t\right)-E_{Rb}\left(t\right).$$

The attacker experiences a loss of the resources utilized due to the attack’s lack of success. Equation (13) provides the calculation for the attacker’s payout:

(13) $$U_{11}\left(A\right)=-R_{CA}\left(t\right).$$

Each approach in this framework is complemented by matching payoffs, demonstrating the balance between the costs and advantages of the chosen defensive and offensive strategies. The comprehensive nomenclature of the parameters used in this framework is shown in Table 8 below.

The utility matrices $\left(M_{D}\right)$ and $\left(M_{A}\right)$ in our cybersecurity game model represent the strategic interaction between the defender and attacker. Positive values $G_{SD}$ and $G_{SA}$ represent the advantages obtained from successful defense and attack, respectively; on the other hand, the costs $E_{Rb}$, $E_{Ab}$, $E_{HNB}$, $R_{CA}$, and $W_{CA}$ have a negative impact on utility, indicating a balance between possible benefits and their associated costs. To calculate the game matrix, we can use the provided strategies and payoffs in the following manner:

- **Matrix for the defender’s payoff ($M_{D}$)**
  $$M_{D}=\left[\begin{array}{ccc} d_{11} & d_{12} & d_{13}\\ d_{21} & d_{22} & d_{23}\\ d_{31} & d_{32} & d_{33} \end{array}\right]=\left[\begin{array}{ccc} G_{SD}\left(t\right)-E_{Rb}\left(t\right) & -E_{Rb}\left(t\right) & -E_{Rb}\left(t\right)-V_{AA}\left(t\right)\\ G_{SD}\left(t\right)-E_{Ab}\left(t\right) & -E_{Ab}\left(t\right) & G_{SD}\left(t\right)-E_{Ab}\left(t\right)\\ -E_{HNB}\left(t\right)-V_{AA}\left(t\right) & -E_{HNB}\left(t\right) & G_{SD}\left(t\right)-E_{HNB}\left(t\right) \end{array}\right]$$
- **Matrix for the attacker’s payoff ($M_{A}$)**
  $$M_{A}=\left[\begin{array}{ccc} a_{11} & a_{12} & a_{13}\\ a_{21} & a_{22} & a_{23}\\ a_{31} & a_{32} & a_{33} \end{array}\right]=\left[\begin{array}{ccc} -R_{CA}\left(t\right) & -W_{CA}\left(t\right) & G_{SA}\left(t\right)-R_{CA}\left(t\right)\\ -R_{CA}\left(t\right) & -W_{CA}\left(t\right) & -R_{CA}\left(t\right)\\ G_{SA}\left(t\right)-R_{CA}\left(t\right) & -W_{CA}\left(t\right) & -R_{CA}\left(t\right) \end{array}\right]$$

We have used the scribing approach [47] to identify the **NE** in analyzing the proposed game-theoretic framework. For this, it is necessary to contrast the optimal function of matrix $M_{A}$ with the equivalent element in matrix $M_{D}$, then identify a solution based on the pure strategies. The attacker’s maximal profits are represented by $a_{13}$ and $a_{31}$; on the other hand, the highest possible profits for the defense are denoted as $d_{11},d_{21},d_{23}$, and $d_{33}$. From the above model, we can draw two important conclusions.

**Theorem 1.**  *There is no **NE** in pure strategies for the suggested game-theoretic model, which is represented by the utility matrices $M_{D}$ and $M_{A}$.*

**Proof.**  This conclusion can be derived from the observation that the attacker’s optimum choices ($a_{13}$ and $a_{31}$ in matrix $M_{A}$) do not correspond to any of the defender’s optimal choices ($d_{11},d_{21},d_{23}$, and $d_{33}$ in matrix $M_{D}$). Given that an **NE** necessitates that each player’s strategy be the optimum response to the other player’s approach, the absence of concurrence between the attacker’s and defender’s optimal strategies suggests that a pure strategy for the **NE** does not exist in this scenario. □

**Theorem 2.**  *The attacker should consistently initiate the attack to optimize the potential gains within the suggested game-theoretic framework.*

**Proof.**  Within the framework of the proposed model, the utility matrices $M_{A}$ and $M_{D}$ are used to assess the attacker’s strategies by considering their prospective benefits and expenses. The utility matrix $M_{A}$ reveals that actions such as $a_{13}$ and $a_{31}$ provide the most benefits for the attacker, as they correspond to situations where the attacker decides to initiate an attack. The benefits of these gains are compared with the disadvantages of waiting or using other techniques that do not include attacking, which tend to be comparatively less advantageous. Given that the attacker’s main goal is to optimize their gains, it is preferable for them to use techniques that result in the maximum level of utility. Hence, based on the configuration of the utility matrix $M_{A}$ and the comparative assessments of the advantages and disadvantages linked to each strategy, it is apparent that engaging in an attack results in the highest benefit for the attacker. Consequently, it can be deduced that the attacker will consistently select an attack to optimize their utility. □

Table 9 shows the results of the above analysis.

As the detection rates of the IDS for Rate-based DDoS, Anomaly-based, and Heuristic Network Behavior attacks are α,β, and γ, respectively, from Table 9 we have the following payoff matrices for the defender and attacker:

$${\tilde{M}}_{D}=\left[\begin{array}{ccc} \alpha G_{SD}\left(t\right)-E_{Rb}\left(t\right)-\left(1-\alpha \right)V_{AA}\left(t\right)\; & \beta G_{SD}\left(t\right)-E_{Ab}\left(t\right)-\left(1-\beta \right)V_{AA}\left(t\right)\; & -E_{HNB}\left(t\right)-V_{AA}\left(t\right)\\ -E_{Rb}\left(t\right)-V_{AA}\left(t\right)\; & \beta G_{SD}\left(t\right)-E_{Ab}\left(t\right)-\left(1-\beta \right)V_{AA}\left(t\right)\; & \gamma G_{SD}\left(t\right)-E_{HNB}\left(t\right)-\left(1-\gamma \right)V_{AA}\left(t\right) \end{array}\right]$$

and

$${\tilde{M}}_{A}=\left[\begin{array}{ccc} \left(1-\alpha \right)G_{SA}\left(t\right)-R_{CA}\left(t\right)\; & \left(1-\beta \right)G_{SA}\left(t\right)-R_{CA}\left(t\right)\; & G_{SA}\left(t\right)-R_{CA}\left(t\right)\\ G_{SA}\left(t\right)-R_{CA}\left(t\right)\; & \left(1-\beta \right)G_{SA}\left(t\right)-R_{CA}\left(t\right)\; & \left(1-\gamma \right)G_{SA}\left(t\right)-R_{CA}\left(t\right) \end{array}\right].$$

To estimate the relevant payoffs for both the defender and the attacker in each of the following scenarios (see Table 10), we use the modified 2×3 matrices, denoted as ${\tilde{M}}_{D}$ and ${\tilde{M}}_{A}$.

Below, we discuss several cases.

**Case 1:**  In the above context, the defender uses the rate-based DDoS and anomaly-based methods with the corresponding probabilities $\eta_{1}$ and $\left(1-\eta_{1}\right)$. Similarly, the probabilities associated with the attacker initiating a volumetric DDoS attack and an RTSP brute-force attack are represented by the corresponding variables $\vartheta_{1}$ and $\left(1-\vartheta_{1}\right)$. Considering the relevant probabilities, the following equations represent the cumulative advantages for the defender (U(D)) and attacker (U(A)):
  $$U\left(D\right)=\eta_{1}\vartheta_{1}U_{11}\left(D\right)+\left(1-\eta_{1}\right)\vartheta_{1}U_{12}\left(D\right)+\eta_{1}\left(1-\vartheta_{1}\right)U_{21}\left(D\right)+\left(1-\eta_{1}\right)\left(1-\vartheta_{1}\right)U_{22}\left(D\right)$$
  and
  $$U\left(A\right)=\eta_{1}\vartheta_{1}U_{11}\left(A\right)+\left(1-\eta_{1}\right)\vartheta_{1}U_{12}\left(A\right)+\eta_{1}\left(1-\vartheta_{1}\right)U_{21}\left(A\right)+\left(1-\eta_{1}\right)\left(1-\vartheta_{1}\right)U_{22}\left(A\right).$$
  The following equations represent the partial derivatives of the payoffs for the defender (U(D)) and attacker (U(A)) with respect to probabilities $\eta_{1}$ and $\vartheta_{1}$, respectively:
  $$\begin{array}{ccc} \frac{\partial U(D)}{\partial \eta_{1}} & = & \vartheta_{1}\left[\left(\alpha -\beta \right)G_{SD}\left(t\right)+\left(E_{Ab}\left(t\right)-E_{Rb}\left(t\right)\right)+\left(\beta -\alpha \right)V_{AA}\left(t\right)\right]\\ & & +\left(1-\vartheta_{1}\right)\left[(\beta G_{SD}\left(t\right)-E_{Ab}\left(t\right)+\beta V_{AA}\left(t\right)-E_{Rb}\left(t\right)-V_{AA}\left(t\right))\right] \end{array}$$
  and
  $$\begin{array}{c} \frac{\partial U(A)}{\partial \vartheta_{1}}=-\eta_{1}\alpha G_{SA}\left(t\right)-\left(1-\eta_{1}\right)\beta G_{SA}\left(t\right). \end{array}$$
  By solving the above equations, we obtain
  (14) $$\begin{array}{ccc} \vartheta_{1} & = & \frac{-\left[\beta G_{SD}\left(t\right)-E_{Ab}\left(t\right)+\beta V_{AA}\left(t\right)-E_{Rb}\left(t\right)-V_{AA}\left(t\right)\right]}{\left[\left(\alpha -2\beta \right)G_{SD}\left(t\right)+2\left(E_{Ab}\left(t\right)-E_{Rb}\left(t\right)\right)+\left(1-2\beta +\alpha \right)V_{AA}\left(t\right)\right]}, \end{array}$$
  (15) $$\begin{array}{ccc} \left(1-\vartheta_{1}\right) & = & \frac{\left[\left(\alpha -\beta \right)G_{SD}\left(t\right)+\left(E_{Ab}\left(t\right)-E_{Rb}\left(t\right)\right)+\left(\alpha -\beta \right)V_{AA}\left(t\right)\right]}{\left[\left(\alpha -2\beta \right)G_{SD}\left(t\right)+2\left(E_{Ab}\left(t\right)-E_{Rb}\left(t\right)\right)+\left(1-2\beta +\alpha \right)V_{AA}\left(t\right)\right]}, \end{array}$$
  (16) $$\begin{array}{ccc} \eta_{1} & = & \frac{\beta }{\alpha -\beta }, \end{array}$$
  (17) $$\begin{array}{ccc} \left(1-\eta_{1}\right) & = & \frac{\alpha -2\beta }{\alpha -\beta }. \end{array}$$
**Case 2:**  In this case, the probabilities $\eta_{2}$ and $\left(1-\eta_{2}\right)$, represent the defender’s utilization of rate-based DDoS and heuristic network behavior techniques, respectively. Additionally, the variables $\vartheta_{2}$ and $\left(1-\vartheta_{2}\right)$ reflect the likelihood of the attacker attempting an RTSP brute-force assault or a volumetric DDoS attack, respectively. The following equations show the resulting benefits for the attacker (U(A)) and defender (U(D)):
  $$U\left(D\right)=\eta_{2}\vartheta_{1}U_{11}\left(D\right)+\left(1-\eta_{1}\right)\vartheta_{2}U_{12}\left(D\right)+\eta_{1}\left(1-\vartheta_{2}\right)U_{21}\left(D\right)+\left(1-\eta_{1}\right)\left(1-\vartheta_{2}\right)U_{22}\left(D\right)$$
  and
  $$U\left(A\right)=\eta_{2}\vartheta_{1}U_{11}\left(A\right)+\left(1-\eta_{1}\right)\vartheta_{2}U_{12}\left(A\right)+\eta_{1}\left(1-\vartheta_{2}\right)U_{21}\left(A\right)+\left(1-\eta_{1}\right)\left(1-\vartheta_{2}\right)U_{22}\left(A\right).$$
  By solving these equations with $\frac{\partial U(D)}{\partial \eta_{2}}=0$ and $\frac{\partial U(A)}{\partial \vartheta_{2}}$, we obtain
  (18) $$\begin{array}{ccc} \vartheta_{2} & = & \frac{-\gamma G_{SD}\left(t\right)+E_{HNB}\left(t\right)-E_{Rb}\left(t\right)}{\alpha G_{SD}\left(t\right)+\alpha V_{AA}\left(t\right)+\gamma G_{SD}\left(t\right)}, \end{array}$$
  (19) $$\begin{array}{ccc} \left(1-\vartheta_{2}\right) & = & \frac{\alpha G_{SD}\left(t\right)+\alpha V_{AA}\left(t\right)+2\gamma G_{SD}\left(t\right)-E_{HNB}\left(t\right)+E_{Rb}\left(t\right)}{\alpha G_{SD}\left(t\right)+\alpha V_{AA}\left(t\right)+\gamma G_{SD}\left(t\right)}, \end{array}$$
  (20) $$\begin{array}{ccc} \eta_{2} & = & \frac{\gamma }{\alpha +\gamma }, \end{array}$$
  (21) $$\begin{array}{ccc} \left(1-\eta_{2}\right) & = & \frac{\alpha }{\alpha +\gamma }. \end{array}$$
**Case 3:**  The defender uses anomaly-based and heuristic network behavior approaches, respectively indicated by the probabilities $\eta_{3}$ and $\left(1-\eta_{3}\right)$. In addition, the variables $\vartheta_{3}$ and $\left(1-\vartheta_{3}\right)$ represent the possibility of the attacker initiating a volumetric DDoS attack or an RTSP brute-force assault, respectively. The benefits to the attacker (U(A)) and defender (U(D)) are shown in the following equations:
  $$U\left(D\right)=\eta_{2}\vartheta_{1}U_{11}\left(D\right)+\left(1-\eta_{1}\right)\vartheta_{2}U_{12}\left(D\right)+\eta_{1}\left(1-\vartheta_{2}\right)U_{21}\left(D\right)+\left(1-\eta_{1}\right)\left(1-\vartheta_{2}\right)U_{22}\left(D\right)$$
  and
  $$U\left(A\right)=\eta_{2}\vartheta_{1}U_{11}\left(A\right)+\left(1-\eta_{1}\right)\vartheta_{2}U_{12}\left(A\right)+\eta_{1}\left(1-\vartheta_{2}\right)U_{21}\left(A\right)+\left(1-\eta_{1}\right)\left(1-\vartheta_{2}\right)U_{22}\left(A\right).$$
  Equations $\frac{\partial U(D)}{\partial \eta_{3}}=0$ and $\frac{\partial U(A)}{\partial \vartheta_{3}}$ can be solved to obtain
  (22) $$\begin{array}{ccc} \vartheta_{3} & = & \frac{\gamma G_{SD}\left(t\right)-E_{HNB}\left(t\right)-\gamma V_{AA}\left(t\right)}{\left(\beta -\gamma \right)G_{SD}\left(t\right)+\left(E_{Ab}\left(t\right)-E_{HNB}\left(t\right)\right)+\left(\gamma -\beta \right)V_{AA}\left(t\right)}, \end{array}$$
  (23) $$\begin{array}{ccc} \left(1-\vartheta_{3}\right) & = & \frac{\beta G_{SD}\left(t\right)-\gamma G_{SD}\left(t\right)+E_{Ab}\left(t\right)-E_{HNB}\left(t\right)+\gamma V_{AA}\left(t\right)-\beta V_{AA}\left(t\right)}{\left(\beta -\gamma \right)G_{SD}\left(t\right)+\left(E_{Ab}\left(t\right)-E_{HNB}\left(t\right)\right)+\left(\gamma -\beta \right)V_{AA}\left(t\right)}, \end{array}$$
  (24) $$\begin{array}{ccc} \eta_{3} & = & \frac{1-\gamma }{\beta -2\gamma +1}, \end{array}$$
  (25) $$\begin{array}{ccc} \left(1-\eta_{3}\right) & = & \frac{\beta -\gamma }{\beta -2\gamma +1}. \end{array}$$

Supposing that the odds for each event are identical, it can be inferred that both the attacker and the defender will obtain the maximum advantages. Based on Cases 1–3, we arrive at the following **NE** solution for the defender $\left(S_{D}\right)$ and attacker $\left(S_{A}\right)$:

(26) $$\begin{array}{ccc} S_{D} & = & \left[\vartheta_{1},\left(1-\vartheta_{1}\right),\vartheta_{2},\left(1-\vartheta_{2}\right),\vartheta_{3},\left(1-\vartheta_{3}\right)\right]\\ & = & \left[\frac{-\left[\beta G_{SD}\left(t\right)-E_{Ab}\left(t\right)+\beta V_{AA}\left(t\right)-E_{Rb}\left(t\right)-V_{AA}\left(t\right)\right]}{\left[\left(\alpha -2\beta \right)G_{SD}\left(t\right)+2\left(E_{Ab}\left(t\right)-E_{Rb}\left(t\right)\right)+\left(1-2\beta +\alpha \right)V_{AA}\left(t\right)\right]},\right.\\ & & \frac{\left[\left(\alpha -\beta \right)G_{SD}\left(t\right)+\left(E_{Ab}\left(t\right)-E_{Rb}\left(t\right)\right)+\left(\alpha -\beta \right)V_{AA}\left(t\right)\right]}{\left[\left(\alpha -2\beta \right)G_{SD}\left(t\right)+2\left(E_{Ab}\left(t\right)-E_{Rb}\left(t\right)\right)+\left(1-2\beta +\alpha \right)V_{AA}\left(t\right)\right]},\\ & & \frac{-\gamma G_{SD}\left(t\right)+E_{HNB}\left(t\right)-E_{Rb}\left(t\right)}{\alpha G_{SD}\left(t\right)+\alpha V_{AA}\left(t\right)+\gamma G_{SD}\left(t\right)},\\ & & \frac{\alpha G_{SD}\left(t\right)+\alpha V_{AA}\left(t\right)+2\gamma G_{SD}\left(t\right)-E_{HNB}\left(t\right)+E_{Rb}\left(t\right)}{\alpha G_{SD}\left(t\right)+\alpha V_{AA}\left(t\right)+\gamma G_{SD}\left(t\right)},\\ & & \frac{\gamma G_{SD}\left(t\right)-E_{HNB}\left(t\right)-\gamma V_{AA}\left(t\right)}{\left(\beta -\gamma \right)G_{SD}\left(t\right)+\left(E_{Ab}\left(t\right)-E_{HNB}\left(t\right)\right)+\left(\gamma -\beta \right)V_{AA}\left(t\right)},\\ & & \left.\frac{\beta G_{SD}\left(t\right)-\gamma G_{SD}\left(t\right)+E_{Ab}\left(t\right)-E_{HNB}\left(t\right)+\gamma V_{AA}\left(t\right)-\beta V_{AA}\left(t\right)}{\left(\beta -\gamma \right)G_{SD}\left(t\right)+\left(E_{Ab}\left(t\right)-E_{HNB}\left(t\right)\right)+\left(\gamma -\beta \right)V_{AA}\left(t\right)}\right] \end{array}$$

and

(27) $$\begin{array}{ccc} S_{A} & = & \left[\eta_{1},\left(1-\eta_{1}\right),\eta_{2},\left(1-\eta_{2}\right),\eta_{3},\left(1-\eta_{3}\right)\right]\\ & = & \left[\frac{\beta }{\alpha -\beta },\frac{\alpha -2\beta }{\alpha -\beta },\frac{\gamma }{\alpha +\gamma },\frac{\alpha }{\alpha +\gamma },\frac{1-\gamma }{\beta -2\gamma +1},\frac{\beta -\gamma }{\beta -2\gamma +1}\right]. \end{array}$$

Our game-theoretic approach focuses on three specific strategies for the attacker and defender. Using this approach, we can depict a more complex model which allows us to find the optimal strategy and Nash equilibrium (**NE**) within the given framework. These methods can be employed to illustrate the complex relationships between the defender and attacker in the proposed Network Intrusion Detection System (IDS) for Internet of Things (IoT) security.

As IoT systems become more complicated and ubiquitous, formal validation methodologies are increasingly important. Formal methodologies employ mathematical models to systematically design, construct, and validate systems to guarantee that they possess the desired characteristics and function efficiently in various environments [48,49]. Despite the dynamic and varied nature of the IoT environment, formal techniques can provide a high level of assurance against safety vulnerabilities and malfunctions. Developers can reduce the probability of significant defects in deployable systems by identifying and fixing potential problems early in the design phase. Model checking, theorem proving, and formal specification languages are among the methods for accomplishing this. It is also possible to improve interactions between interconnected IoT devices by combining game theory with formal techniques. Game theory provides a framework for modeling strategic interactions in IoT environments with competing resources and potentially hostile behaviors. As decisions made by one device impact the results of others, this integration improves the potential of developing resilient and robust IoT systems by ensuring cooperative behavior across devices and anticipating and mitigating conflicts. Consequently, integrating formal methodologies and game theory into the validation process can enhance IoT systems’ robustness, security, and trustworthiness.

The computational complexity of each algorithm is shown in Table 11. The costly terms of the proposed scheme are defined in Algorithms 1, 2, and 3.

Table 12 shows detailed comparisons with existing approaches. Verma et al. [50] examined how machine learning and random search categorization might improve IoT security against DoS attacks. They examined classifiers in depth to improve anomaly-based intrusion detection systems (IDS), assessed their efficacy using key metrics and validation procedures, and compared them on the CIDDS-001, UNSW-NB15, and NSL-KDD datasets. Qiu et al. [51] employed black-box access to generate a novel adversarial assault on a DL-based NIDS in the IoT. They used model extraction with fewer data points and a saliency map to duplicate the model to identify the most influential packet features. Almiani et al. [52] developed a fully automated IDS to improve cloud computing security and avoid cyberattacks. A multi-layered recurrent neural network model is used in cloud computing environments near end users and IoT devices. Anthi et al. [53] proposed a three-layer IDS to identify IoT network intrusions. Their system classifies and profiles IoT device behavior detects malicious packets and identifies attack types. Granjal et al. [54] used anomaly-based intrusion detection to detect DoS and 6LoWPAN/CoAP assaults. According to their experiments, this method provides an effective defense against these threats. Yang et al. [55] presented a lightweight detection technique for FL-based IoT intrusion detection against poisoning attacks. Their scoring method filters out unusual central server actors and their models from the global aggregate based on the size of the dataset and the local model loss. Suge et al. [56] introduced an IDS that uses deep learning and machine intelligence to combat security assaults in IoT networks. It compares attack detection with LSTM and KNN algorithms regarding detection time, kappa statistics, geometric mean, and sensitivity. The efficacy of the IDS was examined using Bot-IoT datasets. Saeed et al. [57] presented a sophisticated security framework utilizing random neural networks (RNNs) for intrusion detection and prevention. Using an instrumented source code analysis technique, this method looks for out-of-bound memory accesses during compilation. Each memory allocation generates its own set of tags and each memory visit adds instructions to verify the tags. Our approach uses the multimodal features with transfer learning, providing excellent classification results.

## 6. Conclusions

IoT applications and resources are vulnerable to flood attacks, especially DDoS attacks. Under such attacks, the resources of the targeted device become inaccessible to authorized users due to the enormous influx of network packets. Because of the complexity of the system and the number of features involved, developing an intelligent IDS is extremely difficult. Moreover, choosing adequate features that capture the distinguishing aspects of attacks while minimizing dimensionality remains a challenge [58,59]. This paper provides an enhanced IDS for IoT security by combining multimodal large data representation and transfer learning. Initially, PCAPs are evaluated to extract relevant attack types and bytes. Following this, Spark-based techniques for optimizing big data are implemented to manage massive amounts of data. The transfer learning process is then used to generate semantically enriched trained features. Finally, combining multimodal features, including training and texture features, improves the classification of various cyberattacks. The CIC-IoT 2022 and CIC-IoT 2023 datasets, two well-known IoT-based datasets, were used to examine the proposed method thoroughly. In addition, a game theory-based methodology was utilized in the validation phase to validate the proposed method’s efficacy formally. Three standard deep learning models, CNN-LSTM, CNN-RNN, and CNN-GRU, were designed for the experiments. The results show that the proposed method using a CNN-LSTM model provides the highest classification results. For instance, the proposed CNN-LSTM model obtained 98.1% precision, 98.4% recall, 97.9% f1-score, and 98.2% accuracy on the CIC-IoT 2022 dataset. On the CIC-IoT 2023 dataset, our CNN-LSTM model obtained 97% precision, 96.1% recall, 96.1% f1-score, and 96.4% accuracy.

Nonetheless, the following limitations and challenges must be addressed before deploying the proposed system in a real setting:

- Adversarial Robustness: Addressing adversarial threats is critical for IDS reliability and efficacy in adversarial circumstances. Detecting and mitigating network traffic data modifications can help to defend the system against sophisticated attackers.
- Privacy-preserving Techniques: An IDS with privacy-preserving methods can reduce concerns about collecting and analyzing sensitive network traffic data. It is possible to protect user privacy and detect threats efficiently using homomorphic encryption, secure multiparty computation, and differential privacy.
- Cross-Domain Generalization: The system’s applicability and efficacy can be expanded across IoT domains and surroundings. The IDS can be trained on various datasets to adapt to deployment scenarios, including IoT applications, topologies, and protocols.
- Energy Efficiency: Improving the system’s energy efficiency is vital, especially in IoT environments where resources are typically constrained. Energy-efficient techniques for data processing, feature extraction, and model inference can minimize IDS computing and energy requirements while maintaining performance.
- Robustness Against Evolving Threats: DDoS attacks and other cyber threats evolve as attackers adopt new techniques and strategies. Advanced techniques can be used to detect and address emerging threats that are not included in training datasets.

## 7. Future Directions

The following prospective research directions could improve the proposed system’s performance in various ways:

- Using Explainable Artificial Intelligence (XAI) and advanced transfer learning techniques with big data technology is an exciting direction. This integration uses smart feature engineering and interpretable models. The resulting strategy promotes confidence and intelligence in big data solutions by focusing on transparency, interpretability, and usability. Future research could look into novel strategies for improving the explainability of complex models while retaining the scalability and effectiveness of big data analytics.
- Research strategies could be formulated for feature extraction algorithms that dynamically adapt to evolving network conditions and attack patterns. Reinforcement learning techniques that can optimize feature extraction in real time to enhance detection accuracy should be investigated.
- The integration of IDS with edge computing frameworks to enhance distributed intrusion detection and response capabilities could allow for evaluating network-edge lightweight feature extraction and model inference to reduce latency and bandwidth.
- Strategies can be designed for detecting and mitigating zero-day threats that exploit previously undiscovered vulnerabilities. Analyzing behavior-based analysis and anomaly detection techniques can help to detect novel attack behaviors without the need to depend on previously identified signatures.
- Profiling IoT devices and analyzing their behavior can reveal security threats. Such methods can aid in constructing detailed device profiles and detecting deviations from usual behavior.

## Figures and Tables

**Table 1:** CNN-LSTM performance metrics on the CIC-IoT 2022 dataset.

Flood Attacks	Precision	Recall	F1-Score
Amcrest	0.95	1.00	0.98
Arlo Basestation Camera	1.00	0.99	1.00
ArloQ Camera	1.00	1.00	1.00
Borun Camera	1.00	0.96	0.98
DLink Camera	1.00	1.00	1.00
HeimVision Camera	1.00	1.00	1.00
Home Eye Camera	1.00	0.99	0.99
Luohe Camera	1.00	1.00	1.00
Nest Camera	1.00	0.98	0.99
Netatmo Camera	0.97	1.00	0.98
SimCam	1.00	1.00	1.00

**Table 2:** CNN-RNN performance metrics on the CIC-IoT 2022 dataset.

Flood Attacks	Precision	Recall	F1-Score
Amcrest	0.88	0.75	0.81
Arlo Basestation Camera	1.00	0.99	1.00
ArloQ Camera	1.00	0.91	0.95
Borun Camera	0.94	0.81	0.87
DLink Camera	1.00	0.86	0.93
HeimVision Camera	0.88	0.70	0.78
Home Eye Camera	1.00	0.95	0.98
Luohe Camera	0.58	1.00	0.74
Nest Camera	0.95	1.00	0.98
Netatmo Camera	0.95	0.94	0.94
SimCam	0.98	1.00	0.99

**Table 3:** CNN-GRU performance metrics on the CIC-IoT 2022 dataset.

Flood Attacks	Precision	Recall	F1-Score
Amcrest	0.88	0.75	0.81
Arlo Basestation Camera	1.00	0.99	1.00
ArloQ Camera	1.00	0.91	0.95
Borun Camera	0.94	0.81	0.87
DLink Camera	1.00	0.86	0.93
HeimVision Camera	0.88	0.70	0.78
Home Eye Camera	1.00	0.95	0.98
Luohe Camera	0.55	0.98	0.71
Nest Camera	0.95	1.00	0.98
Netatmo Camera	0.95	0.94	0.94
SimCam	0.98	1.00	0.99

**Table 4:** CNN-LSTM performance metrics on the CIC-IoT 2023 dataset.

DDoS Attacks	Precision	Recall	F1-Score
SYN_Flood	0.75	0.97	0.85
TCP_Flood	1.00	1.00	1.00
SynonymousIP_Flood	0.96	0.69	0.80
UDP_Flood	1.00	1.00	1.00
ICMP_Flood	1.00	0.99	1.00
PSHACK_Flood	1.00	1.00	1.00
RSTFINFlood	1.00	1.00	1.00
HTTP_Flood	0.98	0.98	0.98
ACK_Fragmentation	0.99	0.99	0.99
ICMP_Fragmentation	0.99	0.99	0.99

**Table 5:** CNN-RNN performance metrics on the CIC-IoT 2023 dataset.

DDoS Attacks	Precision	Recall	F1-Score
SYN_Flood	0.73	0.98	0.83
TCP_Flood	1.00	1.00	1.00
SynonymousIP_Flood	0.96	0.64	0.77
UDP_Flood	1.00	1.00	1.00
ICMP_Flood	1.00	0.99	1.00
PSHACK_Flood	1.00	1.00	1.00
RSTFINFlood	1.00	1.00	1.00
HTTP_Flood	0.99	0.98	0.98
ACK_Fragmentation	0.99	0.99	0.99
ICMP_Fragmentation	0.98	0.99	0.99

**Table 6:** CNN-LSTM performance metrics on the Edge-IIoTse dataset.

Class	Precision	Recall	F1-Score
Backdoor	0.89	0.95	0.93
DDoS HTTP Flood	0.95	0.95	0.96
DDoS ICMP Flood	0.97	0.99	0.96
DDoS TCP SYN Flood	0.94	0.95	0.94
DDoS UDP Flood	1.00	1.00	1.00
MITM (ARP spoofing + DNS)	0.85	0.94	0.9
OS Fingerprinting	0.99	1.00	0.98
Password	0.97	0.81	0.87
Port Scanning	1.00	0.96	0.96
Ransomware	0.97	1.00	0.98
SQL injection	1.00	0.96	0.98
Uploading	0.99	0.98	0.97
Vulnerability scanner	1.00	0.98	0.98
XSS	0.98	1.00	0.97

**Table 7:** Comparison of performance metrics on all three datasets.

Dataset	Method	Precision	Recall	F1-Score	Accuracy
CIC-IoT dataset 2022	CNN-LSTM	0.981	0.984	0.979	0.982
	CNN-RNN	0.958	0.953	0.951	0.954
	CNN-GRU	0.921	0.906	0.897	0.902
CIC-IoT dataset 2023	CNN-LSTM	0.970	0.961	0.961	0.964
	CNN-RNN	0.958	0.963	0.958	0.961
Edge-IIoT dataset	CNN-LSTM	0.965	0.963	0.956	0.962
	CNN-RNN	0.943	0.942	0.936	0.940

**Table 8:** Nomenclature of the parameters used in the proposed game-theoretical model.

Description	Symbol
Energy consumed by Rate-based DDoS IDS	$E_{Rb}$
Energy consumed by Anomaly-based IDS	$E_{Ab}$
Energy consumed by Heuristic Network Behavior IDS	$E_{HNB}$
Gain for successfully detecting	$G_{SD}$
Value of the assets under attack	$V_{AA}$
Resource consumption by attacker	$R_{CA}$
Gain for successfully attacking	$G_{SA}$
Waiting time cost for attacker	$W_{CA}$
Detection rate of IDS for Rate-based DDoS	α
Detection rate of IDS for Anomaly-based	β
Detection rate of Heuristic Network Behavior	γ
False Positive rate of Defender	μ

**Table 9:** Modified utility matrix for the defender and attacker.

Defender $\left(S_{D}\right)$	Rate-Based DDoS IDS	Anomaly-Based IDS	Heuristic Network Behavior IDS
Volumetric DDoS	$G_{SD}\left(t\right)-E_{Rb}\left(t\right)$, $-R_{CA}\left(t\right)$	$G_{SD}\left(t\right)-E_{Ab}\left(t\right)$, $-R_{CA}\left(t\right)$	$-E_{HNB}\left(t\right)-V_{AA}\left(t\right)$, $G_{SA}\left(t\right)-R_{CA}\left(t\right)$
RTSP Brute-Force	$-E_{Rb}\left(t\right)-V_{AA}\left(t\right)$, $G_{SA}\left(t\right)-R_{CA}\left(t\right)$	$G_{SD}\left(t\right)-E_{Ab}\left(t\right)$, $-R_{CA}\left(t\right)$	$G_{SD}\left(t\right)-E_{HNB}\left(t\right)$, $-R_{CA}\left(t\right)$

**Table 10:** Different scenarios and relevant defender’s strategies.

Cases	Defender’s Strategies
Case 1	Rate-Based DDOS or Anomaly-Based
Case 2	Rate-Based DDOS or Heuristic Network Behavior
Case 3	Anomaly-Based or Heuristic Network Behavior

**Table 11:** Complexity analysis.

Cost Terms	Algorithm 1	Algorithm 2	Algorithm 3
Ini	|n|	2|n|	−
E,Co	|n|	2|n|	2|n|
D,f	−	2|n|+3|ResNet|	5|f|
ρ,σ,μ	−	−	$|\rho_{t}\left|+3|E\right|$
Total cost	2|n|	6|n|+3|ResNet|	$2|n|+5|f|+|\rho_{t}\left|+3|E\right|$

Ini stands for Initialization, *E* for encryption, Co for computeness, *D* for decomposition, *f* for functions, ResNet for residual network functions, ρ for DAGScheldular, μ for derive process, and σ for Spark yarn client.

**Table 12:** Performance comparisons with existing methods.

Work	Method	Accuracy
Verma et al. [47]	Random Search with ML	0.967
Qiu et al. [48]	Adversarial DNN	0.943
Almiani et al. [49]	Deep RNN	0.924
Anthi et al. [50]	Supervised ML	0.98
Granjal et al. [51]	SVM with Kernels	0.933
Yang et al. [52]	Federated Learning	0.971
Suge et al. [56]	LSTM	0.973
Saeed et al. [57]	Random Neural Networks	0.972
Our Method	Multimodal with Transfer Learning	0.982

## Data Availability

The data that support the findings of this study are openly available in the Canadian Institute for Cybersecurity CIC-IoT 2022 and CIC-IoT 2023 datasets at https://www.unb.ca/cic/datasets/iotdataset-2022.html and https://www.unb.ca/cic/datasets/iotdataset-2023.html, respectively (accessed on 30 October 2023).
